# Genome defense against integrated organellar DNA fragments from plastids into plant nuclear genomes through DNA methylation

**DOI:** 10.1038/s41598-019-38607-6

**Published:** 2019-02-14

**Authors:** Takanori Yoshida, Hazuka Y. Furihata, Taiko Kim To, Tetsuji Kakutani, Akira Kawabe

**Affiliations:** 1 Faculty of Life Science, Kyoto Sangyo University, Kyoto, Kyoto Japan; 2 Faculty of Science, The University of Tokyo, Bunkyo-ku, Tokyo Japan; 3 Department of Integrated Genetics, National Institute of Genetics, Mishima, Shizuoka Japan; 4 Department of Genetics, School of Life Science, The Graduate University for Advanced Studies (SOKENDAI), Mishima, Shizuoka Japan

**Keywords:** Evolutionary genetics, DNA methylation, Plant evolution

## Abstract

Nuclear genomes are always faced with the modification of themselves by insertions and integrations of foreign DNAs and intrinsic parasites such as transposable elements. There is also substantial number of integrations from symbiotic organellar genomes to their host nuclear genomes. Such integration might have acted as a beneficial mutation during the evolution of symbiosis, while most of them have more or less deleterious effects on the stability of current genomes. Here we report the pattern of DNA substitution and methylation on organellar DNA fragments integrated from plastid into plant nuclear genomes. The genome analyses of 17 plants show homology–dependent DNA substitution bias. A certain number of these sequences are DNA methylated in the nuclear genome. The intensity of DNA methylation also decays according to the increase of relative evolutionary times after being integrated into nuclear genomes. The methylome data of epigenetic mutants shows that the DNA methylation of organellar DNA fragments in nuclear genomes are mainly dependent on the methylation maintenance machinery, while other mechanisms may also affect on the DNA methylation level. The DNA methylation on organellar DNA fragments may contribute to maintaining the genome stability and evolutionary dynamics of symbiotic organellar and their host’s genomes.

## Introduction

Recently, it is recognized that epigenetic mechanisms are important for regulation of foreign DNAs that are integrated into host genomes and disrupt genome stability. For example, regulation of transposable elements (TEs) is important in maintaining genome stability. In general, the mobility of TEs in plants is highly repressed by multiple factors. One of the important mechanisms involved in genome stability and repression of endogenous TEs and foreign DNA fragments is RNA interference (RNAi). RNAi participates in various nuclear processes such as repression of TEs, heterochromatin formation, and gene expression regulation^1,2^. Such phenomena are caused by post–transcriptional gene silencing (PTGS) and epigenetic modifications such as DNA methylation by RNA–directed DNA methylation (RdDM) and histone modification. In addition to RdDM, DNA methylation is maintained by methylation maintenance machinery with DNA methyltransferase that propagate CpG methylation on newly synthesized DNA and non-CpG DNA and histone methyltransferases involved in the crosstalk of DNA and histone methylation^3–9^. In plant, the existence of another pathway that leads to *de novo* DNA methylation without small RNA is also suggested by analyses of mutants of the *DDM1* (*DECREASE IN DNA METHYLATION 1*) gene in the model plant *A*rabidopsis *thaliana*^10^. Since the discovery of mobile elements in maize^11,12^, TEs were mainly considered as ‘selfish DNA’ that has little functional or beneficial effects to host genomes^13^. In recent years, this classical view has been challenged by a new hypothesis that TEs contribute to the expansion of genome complexity in eukaryotes^14–16^. Epigenetic modifications such as histone modification and DNA methylation on endogenous TEs and exogenous invading DNAs can be thus important not only for the repression of such sequences, but also for gene regulations, expression networks and genome evolvability.

Organellar genomes also affect the complexity of plant nuclear genomes. Plastid and mitochondrion are important plant organelles that have their own genomes. During the evolution of plastid and mitochondrion, substantial gene transfers from organelle to host nuclear genomes had occurred^17^. The plant genome projects revealed that organellar genomes integration to host nuclear genomes is not limited during the establishment of symbiosis: the organelle DNA fragments from both plastid and mitochondrial genomes are still actively integrated into nuclear genomes and are so–called nuclear plastid DNA (NUPT) and nuclear mitochondrial DNA (NUMT)^18,19^. In *A*. *thaliana*, almost whole mitochondrial genome is integrated into chromosome 2 in relatively recent era^20,21^. In *Oryza sativa*, the extensive study of NUPTs organization suggested recurrent integrations from plastid genomes to nuclear genome, and rapid fragmentation and elimination of NUPTs after integration^22^. NUPTs are also retained in several plant nuclear genomes^23–25^. During the process of integration and elimination of organelle DNA fragments, DNA methylation might play an important role to regulate NUPTs. NUPTs were shown to have excessed substitutions from cytosine (C) to thymine (T) and guanine (G) to adenine (A)^26,27^. These biased substitutions might be induced by hypermethylation of integrated DNAs and subsequent deamination of 5-methylcytosine^17^, suggesting the importance of epigenetic regulation of integrated DNAs. Because the amount of NUPTs (lower than 1% of plant nuclear genomes)^25^ is smaller than that of TEs and the evolutionary change since integrating in nuclear genomes can be estimated by comparing with organellar genomes, the integration and subsequent nucleotide change can be detected easier than those of TEs. Thus, the NUPTs are good materials to investigate the epigenetic mechanism regulating foreign DNA fragments in plant nuclear genomes.

In this study, we investigated the nucleotide changes and DNA methylation status of NUPTs. There are strong biased nucleotide changes correlate with time of integration. By the biased mutation, guanine and cytosine residues compositions changed along with biased substitutions. A certain number of NUPTs are DNA methylated in the nuclear genome. We also detected decay of CpG and non–CpG methylation through time after integration in nuclear genomes. The results in this study suggested that the DNA methylation on NUPTs was mainly dependent on the methylation maintenance machinery, while some NUPTs were more affected by RdDM machinery. Some observations might imply another mechanism of homology-dependent DNA methylation. The mechanism might have roles to defend plant nuclear genomes from deleterious integrations of foreign sequences.

## Results

### Biased mutation in NUPTs

Genome sequence data of 17 plants (13 Eudicots: *A. thaliana, Carica papaya, Vitis vinifera, Lotus japonicus, Medicago truncatula, Glycine max, Manihot esculenta, Ricinus communis, Populus trichocarpa, Cucumis sativus, Fragaria vesca, Solanum tuberosum, Solanum lycopersicum*, 4 Monocots: *Brachypodium distachyon, O. sativa, Sorghum bicolor, Zea mays*) were obtained from databases (Table S1). Integrated NUPTs within each nuclear genome were estimated by BLAST search and subsequent filtering steps. Nucleotide changes between plastid genomes and NUPTs were then identified for each species. To estimate the relative time of integration, the genetic distances (based on AT transversion) between plastid genomes and NUPTs were calculated. NUPTs were grouped by genetic distances (young aged NUPTs; 0.00 to 0.02, middle aged NUPTs; 0.02 to 0.04, old aged NUPTs; 0.04 to 0.06) to characterizing them as a function of integration time.

**Figure 1:**
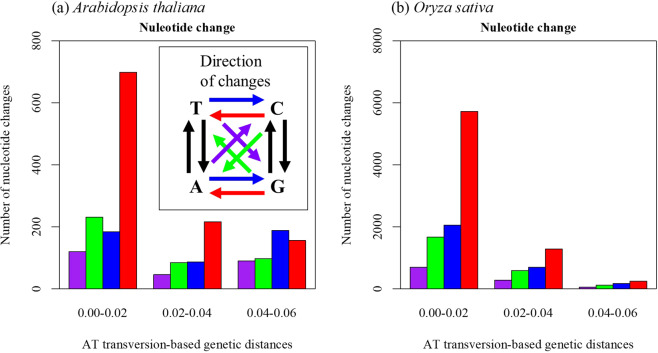
Difference of nucleotide change by integration time. Nucleotide change between NUPTs and corresponding plastid sequences were counted. NUPTs were grouped by AT transversion-based genetic distance that represents timing of integration. Arrows in box represents direction of nucleotide change: red bar; C to T and G to A, blue bar; T to C and A to G, green bar; C to A and G to T, and purple bar; A to C and T to G.

For all species analyzed, strong biased mutations from C/G to T/A were observed as previously suggested^26,27^. These biases were mainly due to the excess of C to T and G to A transitions. The excess of C/G to T/A transition mutations implied that such bias occurred in association with DNA methylation of cytosine residues. The degree of such bias changed by the genetic distance between plastid genome sequences and NUPTs (Fig. 1). In *A*. *thaliana*, the ratio of T/A→C/G transitions to C/G→T/A transitions (i.e., $\frac{{N}_{T\to C}+{N}_{A\to G}}{{N}_{C\to T}+{N}_{G\to A}}$, *N*; the number of transition) was 0.26 at young aged NUPTs. In contrast, the ratio was nearly equal to 1 (1.21) at old NUPTs, suggesting a weakening of biased mutations depending on time after integration. Almost all species analyzed showed a similar pattern of alteration in substitution biases (Fig. S1, Table S2), suggesting a mechanism broadly common in higher plant species.

### Nucleotide composition and patterns of integrated regions of NUPTs

The GC content of NUPTs was decreased in middle aged NUPTs (0.02 to 0.04, Table S2). The decrease of GC contents is consistent with the highly biased transitions at smaller genetic distances (shown in Fig. S1). The differences of GC contents between plastid genomes and NUPTs could be due to reduced G/C residues in majority of DNA fragments (Fig. S2). Compared with younger NUPTs, older NUPTs showed stronger correlation of GC–contents between NUPTs and its flanking regions in *A*. *thaliana* (Fig. S3). It may suggest that at least in *A*. *thaliana*, younger NUPTs are distinguishable from its flanking region by GC–content or sequence pattern of integrated DNA. Although *O*. *sativa* also showed correlation of GC–contents between NUPTs and its flanking regions, the relationship was weaker than that of *A*. *thaliana*. The gene abundance in flanking region of NUPTs (the number of genes in 50 Kb 5′ and 3′ flanking regions) was also surveyed (Fig. S4A). In *A*. *thaliana*, the gene abundance around young NUPTs (median: 18 genes/flanking region) was lower than that of older NUPTs. The density was then shifted towards average value of whole genome gene density (23 genes). There was no significant difference between gene abundance of young and older NUPTs. In *O*. *sativa*, medians of each integration time had no significant change and these values were similar to whole genome gene density (12 genes). Distances from nearest–neighbor gene showed no significant change among groups in both species (Fig. S4A). These results indicated that integrated NUPTs are eliminated somewhat regardless of gene density of its surrounding region.

### Levels of CpG and non–CpG methylation in NUPTs

**Figure 2:**
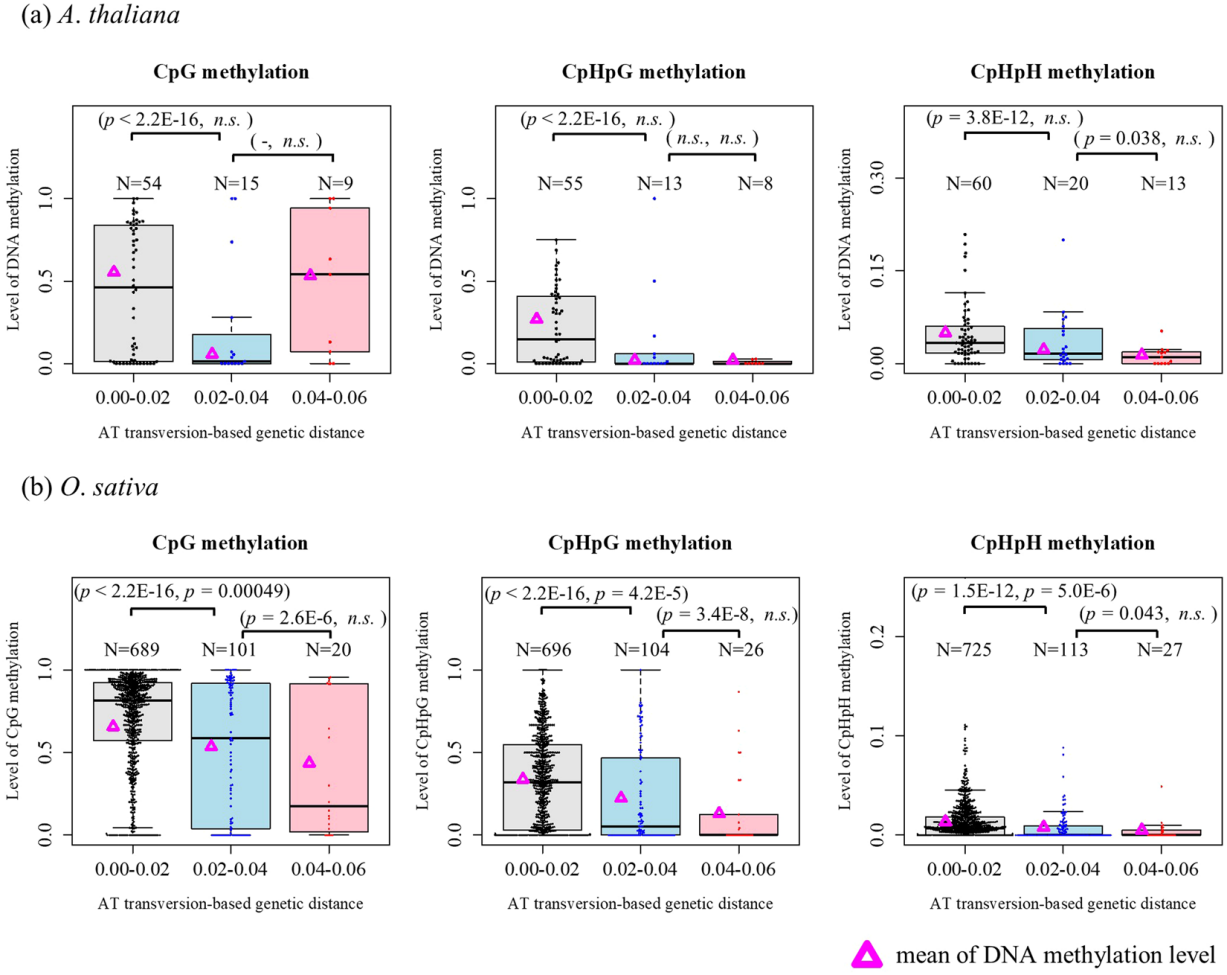
Comparison of DNA methylation level by integration time. Levels of DNA methylation at NUPTs were calculated for CpG, CpHpG and CpHpH sites and were plotted as dots and boxplots. The level of DNA methylation in NUPTs grouped by AT transversion-based genetic distance were plotted: black box/gray dots; small genetic distance (0.01 to 0.02), light blue box/blue dots; medium genetic (0.02 to 0.04), and pink box/red dots; (0.04 to 0.06). The mean of DNA methylation level was plotted by magenta triangles. The significance of statistical tests were shown in parentheses (Χ^2^ test for methylated read counts, Wilcoxon rank sum test for distribution). *n*.*s*.; not significant. N is the number of NUPTs in each CpG/CpHpG/CpHpH dataset. NUPTs with no cytosine site of CpG/CpHpG/CpHpG context in each dataset were excluded from their plotting.

The levels of both CpG and non–CpG methylations for NUPTs were shown in Fig. 2. Interestingly, the levels of DNA methylation showed significant changes through their genetic distances. The levels of cytosine methylation in *O*. *sativa* were time-dependently decreased in both CpG and non–CpG motifs. In *A*. *thaliana*, DNA methylation of non–CpG methylation showed similar pattern of reduction. The methylation level of CpG sites at old aged NUPTs was, however, increased compared with middle aged NUPTs. Because the number of older NUPTs was small (N = 9), this increase of average CpG methylation was predominantly due to the presence of outliers that were strongly methylated (Fig. S5).

The highly methylated fragments with large genetic distances were also observed in *O*. *sativa* (Fig. S5). Most of such NUPTs were located on nearby the coding regions or within the intron of a gene (Table S3), suggesting effects of adjacent regions on the level of CpG methylation of NUPTs. Some older NUPTs would have remained hypermethylated because they are integrated too close to functional genes and hypermethylation of these NUPTs might be necessary for stable expression of adjacent genes.

**Figure 3:**
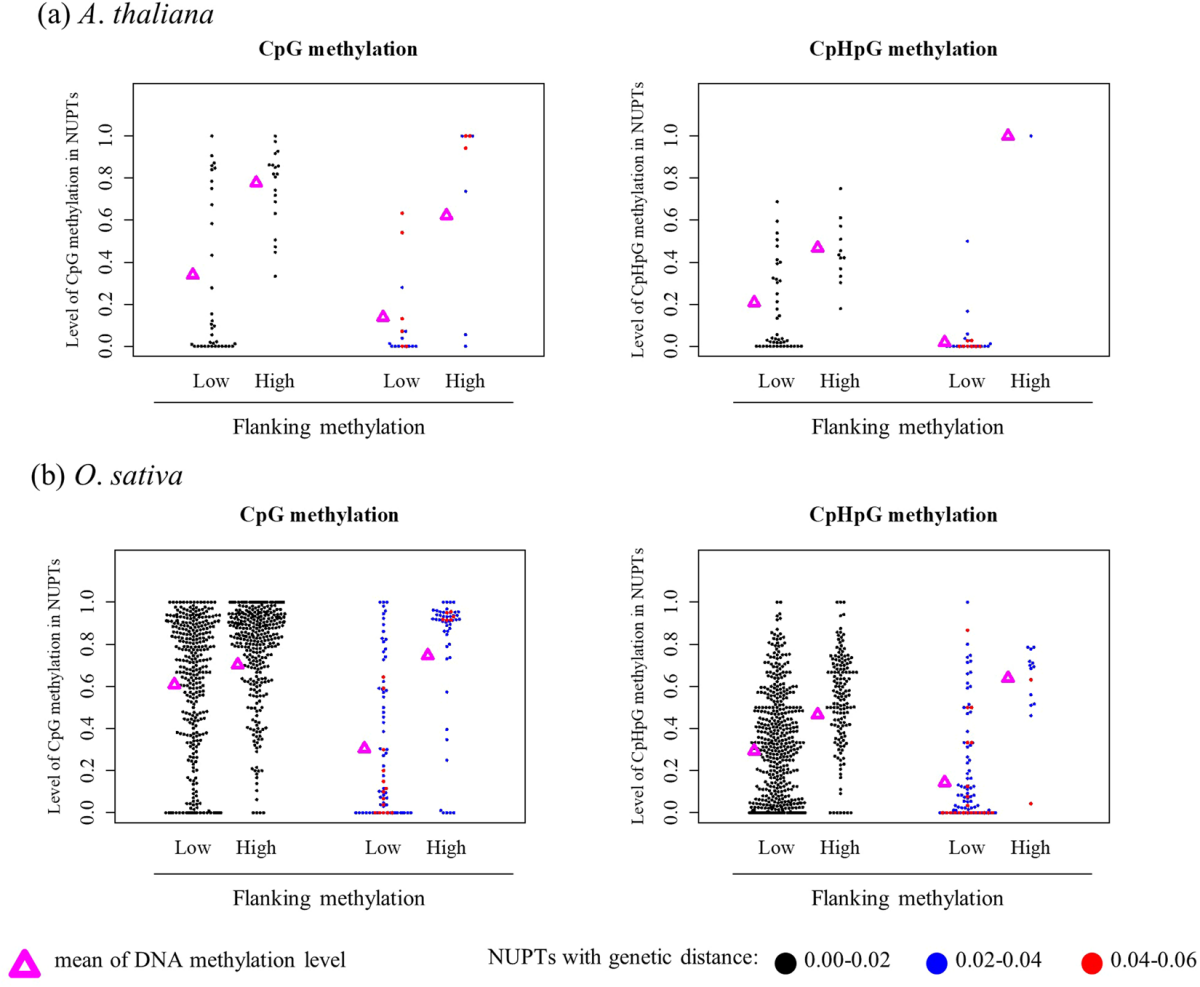
Possible effect of DNA methylation in flanking regions on NUPTs methylation. Adjacent sequences (1 Kb each) from both 5′ and 3′ flanking region of NUPTs were analyzed. Horizontal line represents a level of DNA methylation in flanking region: Low; DNA methylation level <0.5, High; DNA methylation level >= 0.5. Vertical line represents a level of DNA methylation in NUPTs. The mean of DNA methylation level was plotted by magenta triangles.

The levels of DNA methylation at NUPTs were positively correlated with those at flanking sequences, especially in non–CpG methylations (Figs 3 and S6).

The DNA methylation level of NUPTs with larger genetic distance (middle and old aged NUPTs) was strongly associated with that of 5′ and 3′ flanking regions. In contrast, there were highly methylated NUPTs in the group of young aged NUPTs, regardless of low methylation level in flanking regions. The result indicates that the methylation status of older NUPTs depend on the same factor that decides the methylation status of their flanking regions. In contrast, the methylation status of young aged NUPTs is somewhat independent of that of their flanking regions and would be influenced by different factor(s). By these reasons, the methylation status of NUPTs would become similar to that of flanking regions according to integration time.

The observed pattern of DNA methylation levels (Fig. 2) indicates the sequence homology-dependent DNA methylation between NUPTs and their original plastid genome sequences, especially for younger NUPTs (Figs 3 and S6). Alternatively, the correlation between DNA methylation and the genetic distances may simply reflect the elimination of the highly methylated NUPTs; after new integrations, highly methylated NUPTs are predominantly eliminated from nuclear genomes. If it was true, newly integrated NUPTs would be hypermethylated not because of its homology to original plastid genome sequences, but because of its integrated location, GC–content, etc.

### DNA methylation in mutant lines

**Figure 4:**
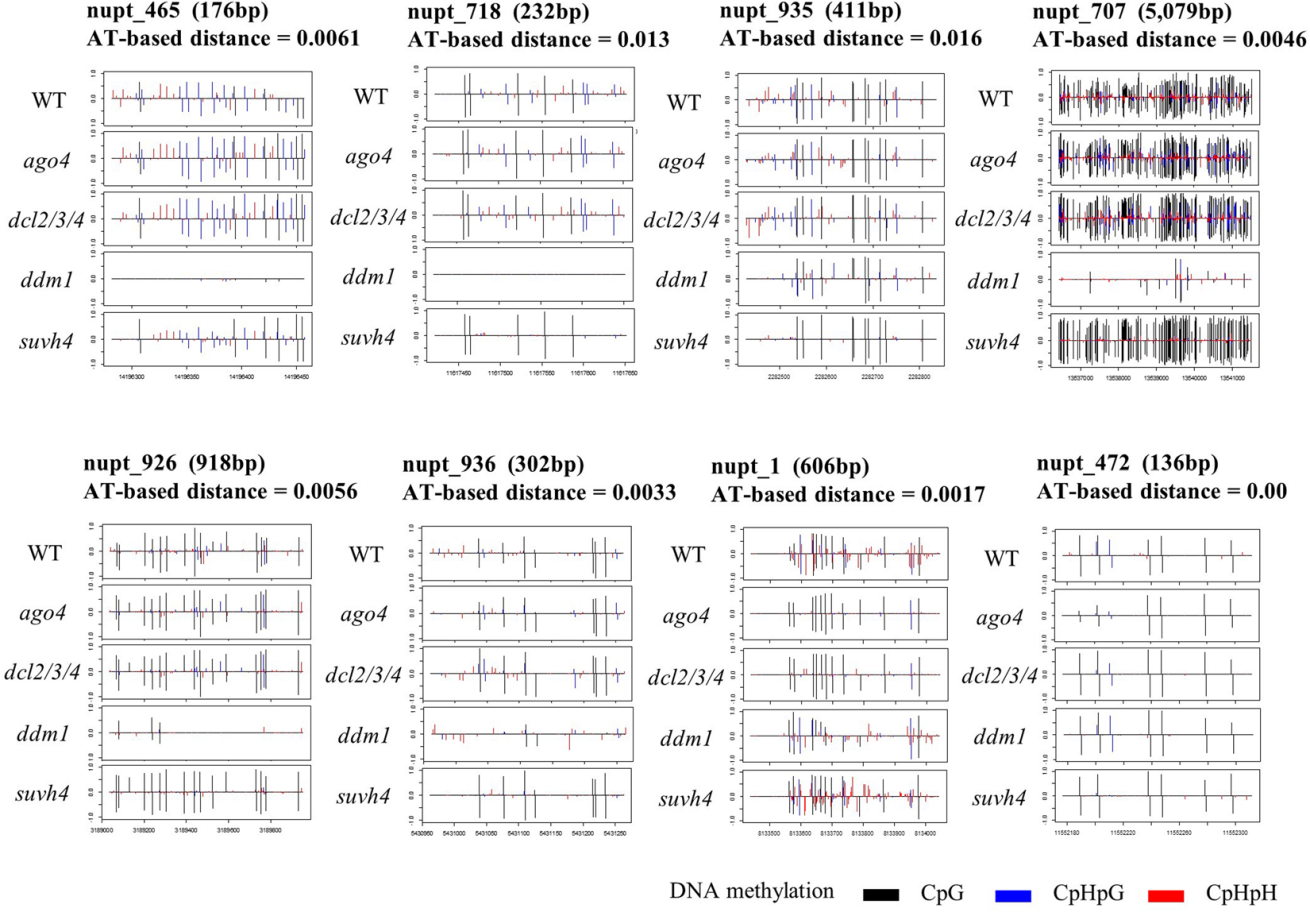
Status of DNA methylation in epigenetic mutant lines. Methylation status by whole genome methylome data. Proportion of CpHpG methylation in 8 representative NUPTs were shown. The sequence length and AT transversion-based genetic distance were also shown for each NUPT. DNA methylation level were shown by colored bars at each site: black; CpG methylation, blue; CpHpG methylation, red; CpHpH methylation.

The DNA methylation in NUPTs could have a similar role as that in defense against foreign DNAs and endogenous TEs. In *A*. *thaliana*, DNA methylation is established and maintained by several methyltransferases: *DNA Methyltransferase 1* (*MET1*), *Chromomethylase 3* & 2 (*CMT*3, *CMT2*), and *Domains Rearranged Methyltransferase 2* (*DRM2*)^28,29^. *DRM2* and other factors such as *Argonaute* 4 (*AGO4*) and *Dicer-like 2/*3*/*4 (*DCL2*/3/4) are involved in the RdDM machinery to repress TEs activity^30^. In addition, there is a positive feedback between histone H3K9 methylation by *SU(VAR)3–9 HOMOLOG 4*/*KRYPTONITE* (*SUVH4/KYP*) and CpHpG and CpHpH methylation by *CMT3* and *CMT2*^9^. In *A*. *thaliana* genome wide methylome data from mutant lines of epigenetic regulation genes are available^31^ that allows us to determine possible mechanisms determining methylation status of NUPTs. In mutant lines of *AGO4* and *DCL2*/*3*/*4*, which might relate to RdDM machinery, many integrated NUPTs except 2 fragments showed no decrease of methylated CpG and non–CpG sites. In contrast, most NUPTs showed hypomethylated cytosines in *ddm1* mutant line (Figs 4 and S7). The results suggest that chromatin remodeling is associated with the maintenance of DNA methylation in NUPTs. Hypomethylations were also shown in both *suvh4*/*kyp*, *cmt3*, and *cmt2* mutants. The exceptional 2 NUPTs (nupt_1, nupt_472) showed loss of methylation in *ago4* and *dcl2*/*3*/*4* suggesting relation to RdDM–dependent methylation in these NUPTs. There are some TEs close to the 2 exceptional NUPTs (Table S4) that could affect the methylation pattern of adjacent NUPTs.

**Figure 5:**
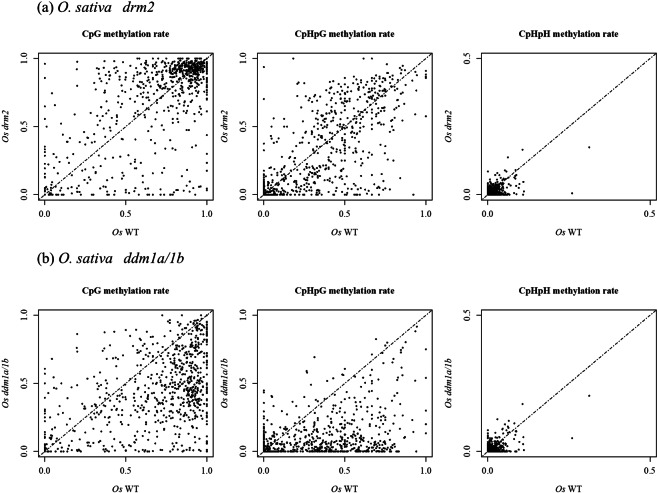
DNA methylation of *O*. *sativa drm2* and *ddm1a/1b* mutants. DNA methylation levels were plotted for *Osdrm2* (**a**) and *Osddm1a/1b* (**b**). Horizontal line represents the level of DNA methylation in WT. Vertical line represents the DNA methylation level of mutant line.

**Figure 6:**
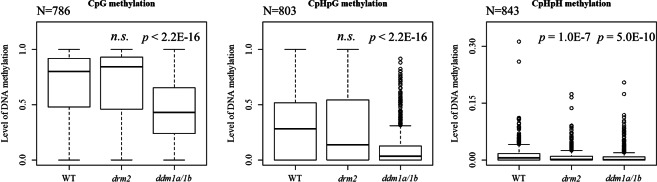
Comparison of DNA methylation level between *O*. *sativa* WT and mutants. Vertical line represents levels of DNA methylation in CpG, CpHpG and CpHpH contexts. Bold line represents medians. Upper and lower lines of boxes represent the first and third quartiles. Circle represents outliers. Wilcoxon rank sum test was conducted. *n*.*s*.; not significant.

In *O*. *sativa*, *Osddm1a/1b* and *Osdrm2* whole-genome bisulfite-seq data^32,33^ were analyzed to verify the tendency observed in *A*. *thaliana*. Compared to *Osdrm2*, *Osddm1a*/*1b* showed more DNA hypomethylation of NUPTs especially for CpG and CpHpG contexts (Fig. 5). In all contexts, there were significant decreases of DNA methylation in *Osddm1a*/*1b* compared to DNA methylation in WT (Fig. 6), indicating that the DNA methylation of *O*. *sativa* NUPTs is also associated with chromatin remodeling factor as observed in *A*. *thaliana*. There were also significant decreases of CpHpH methylation in *Osdrm2*. In contrast, there was no significant difference between WT and *Osdrm2* CpG and CpHpG methylation (Fig. 6).

### TE abundance in flanking regions of NUPTs

**Figure 7:**
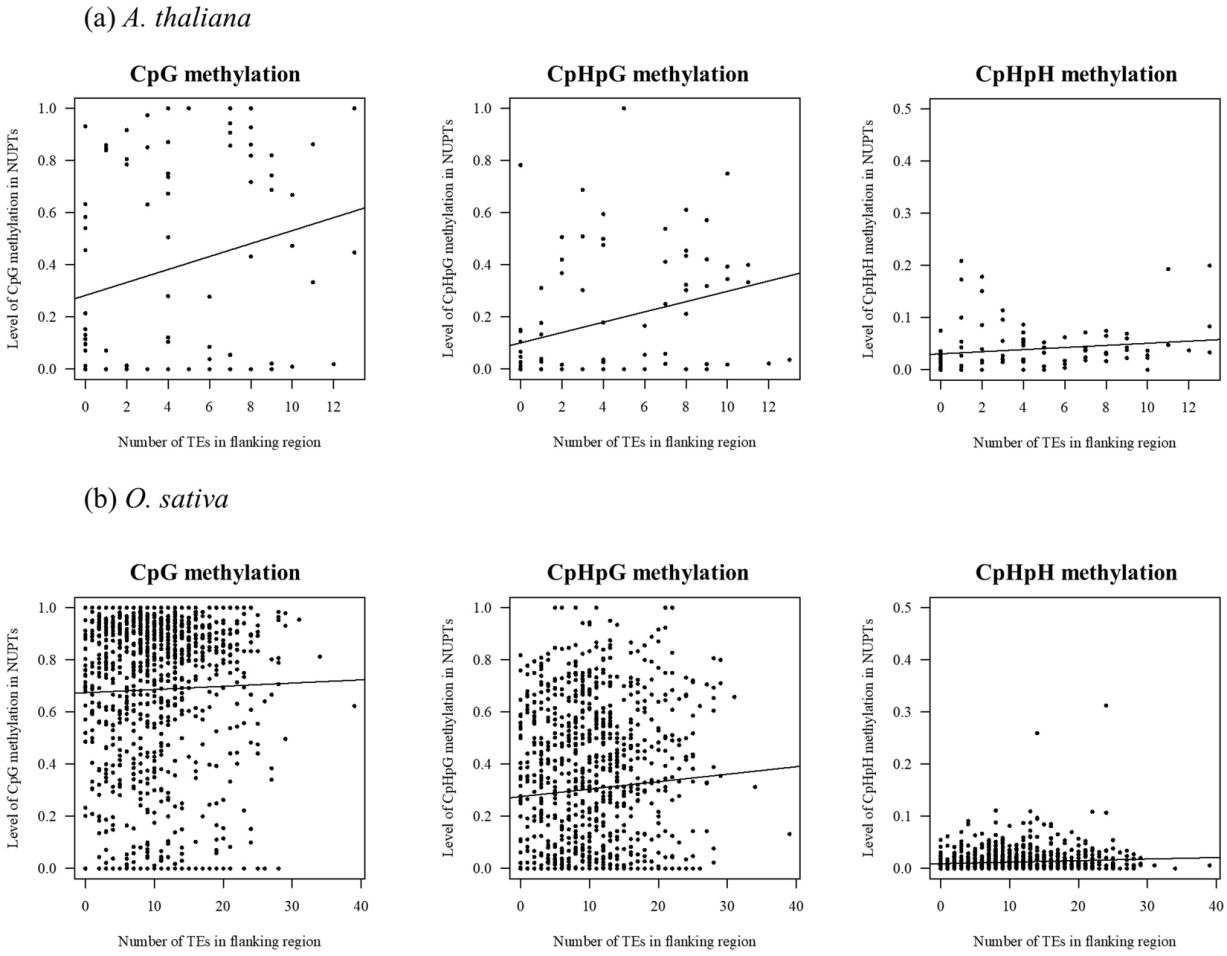
Relationship between levels of NUPT DNA methylation and TE contens in flanking regions. Horizontal line represents the number of TEs in 5 Kb each 5′ and 3′ flanking region. Vertical line represents level of DNA methylation. Solid line represents regression line for CpG, CpHpG, and CpHpH contexts.

The difference of DNA methylation observed within NUPTs could be affected by the TE contents nearby NUPTs. To test the relationship between TEs contents and methylation level, we examined the TE abundance of 5 Kb 5′ and 3′ flanking regions (in total 10 Kb) for *A*. *thaliana* and *O*. *sativa* (Fig. 7). In *A*. *thaliana*, there was positive relationship between number of TEs and CpG/CpHpG methylation level (Pearson’s *ρ* = 0.2312, *p* = 0.032 for CpG, *ρ* = 0.3055, *p* = 0.0050 for CpHpG, Fig. 7a), while there was no significant correlation with CpHpH methylation level (*ρ* = 0.1627, *p* = 0.10). In *O*. *sativa*, there was significant correlation between the TE number and CpHpG/CpHpH methylation level though the correlation coefficients were small (*ρ* = 0.06560, *p* = 0.032 for CpHpG, *ρ* = 0.09323 *p* = 0.0019 for CpHpH, Fig. 7b). The correlation of the TE number with CpG methylation level was not significant (*ρ* = 0.02522, *p* = 0.42). Although the result suggests positive correlation between TE contents in flanking regions and DNA methylation of NUPTs to some extent, the correlation coefficients are relatively small in both species.

### Screening of mutant lines showing hypomethylated organellar DNA-like fragments

The methylome data analyses of *A*. *thaliana* epigenetic regulation gene mutants revealed genetic regulation on the methylation pattern of NUPTs. To elucidate the regulation pathways, over 5 thousand of EMS mutagenized lines were screened for the loss of NUPT DNA methylation by methylation-dependent endonuclease associated PCR (McrBC-PCR).

From the screening, 6 lines showed decrease of DNA methylation on organellar DNA-like fragments. Known genes involved in DNA methylation were checked for mutation within coding region by Sanger sequencing. All of 6 mutant lines had point mutations within coding regions of *DDM1*. In the analysis of mutant methylome data, *ddm1* single mutant shows significant decrease of DNA methylation in NUPTs (Figs 4 and S7), suggesting the strong effect of *ddm1* mutant on NUPTs’ methylation. Although our screening method could successfully detect a single mutant with strong effect, i.e. *ddm1*, the screening did not reveal the novel factor involved in DNA methylation of NUPTs. It may suggest the complexity of the regulation mechanism of NUPTs’ DNA methylation (e.g., the multiple factors and/or lethality of mutants in genes involved), rather than the presence of single responsible gene, and our screening method thus could not detect these potentially complex factors.

## Discussion

After integration, NUPTs are unstable and are eliminated from host nuclear genome^34^. The observation suggests that most *de novo* NUPTs and NUMTs are to some extent selectively deleterious^25,35^. Our results suggest the epigenetic modification by DNA methylation against integrated NUPTs. DNA methylation observed in NUPTs may act as a genome defense for foreign DNA. This hypothetical genome defense against NUPTs by hypermethylation of NUPTs after integration may be partly sequence homology–dependent DNA methylation mechanism (Figs 2 and S5). The genome defense depending on sequence similarity is consistent with our previous results; most new integrations are deleterious and rapidly eliminated, while long–existing older NUPTs are less deleterious and have acquired mutations to became less homology^25^.

We observed decreased GC content of middle aged NUPT compared to young aged NUPTs in all 17 species (Fig. S2). In *A*. *thaliana*, the increase of GC content in old aged NUPTs was also observed (Table S2). This pattern is similar to the change of GC content in TEs over time observed in *A*. *thaliana* genome^36^. This observation could be explained by two mutation bias. While the younger TEs are under C→T mutational force by DNA methylation and succeeding deamination, sufficiently older TEs become out of the target of the DNA methylation machinery and are under influence of the basal mutation bias^36^. Interestingly, while some species showed similar pattern of GC content change (*G*. *max*, *F*. *vesca*, *S*. *tuberosum*), the other species did not show the increase of GC content in old aged NUPTs (Table S2), implying the difference of the basal mutation bias or targeting mechanism for DNA methylation among species.

The results in this study revealed that *DDM1*, *CMT3*, *CMT2*, and *SUVH4*/*KYP* (Figs 4–6 and S7) affect the status of methylation in NUPTs. If most NUPTs cause deleterious effects on genome stability, they should be silenced and tightly-controlled like endogenous TEs. A major factor involved in repressing TEs in plants is DNA methylation. DNA methylation can be *de novo* induced by small interfering RNA^2^. In addition, the DNA methylation can be maintained by DNA methyltransferases *MET1*, and the positive feedback of *CMT3/2* and *SUVH4*. For silencing of inserted DNAs in plant genomes, the feature of insertion sites such as genes and TEs abundance in those regions are important. The position effect and different mechanisms silencing the TEs are well documented for plant genome in which different mechanisms act on TEs inserted in different locations on the genome such as near genes, within genes, pericentromere, and centromeric regions^37^. RdDM machinery is an important mechanism establishing DNA methylation to make the boundary of TEs in genic regions, while *CMT3/2* and *SUVH4* act on maintaining such DNA methylation^5,29,31^. In TE-rich regions such as pericentromeric region and heterochromatic knobs, chromatin remodeler *DDM1* is involved in keeping TEs silenced^29^. The integrated NUPTs could be recognized and become the target of repression by such silencing mechanisms in different regions of nuclear genomes. Alternatively, the NUPTs are methylated by the effect of adjacent TEs in flanking regions in different manner of each region of the genome. The observation that the level of DNA methylation in NUPTs is related to the level of their flanking regions especially for older NUPTs indicates the effect of flanking regions on DNA methylation level (Fig. 3). There were weak but significant correlation between methylation level and TE contents in flanking regions (Fig. 7), suggesting the effect of TE silencing may be also elongated to the NUPTs. The methylome data showed significant decreases of CpHpH methylation in *ago4* and *Osdrm2* mutants (Figs 6 and S7), suggesting that a certain number of NUPTs in addition to 2 examples in Fig. 4 are affected by RdDM machinery in both *A*. *thaliana* and *O*. *sativa*. In previous study of TEs, it is suggested that the DNA methylation of TEs by RdDM machinery can remain active over extended periods of time^36^, suggesting that middle/old aged NUPTs could be also affected by RdDM, as well as young aged NUPTs.

Some observations in this study might have difficulties being explained by the position effect solely. The level of DNA methylation and the rate of C/G to T/A nucleotide changes were decreased according to the genetic distance to the plastid genome sequence (Figs 1, 2 and S1, Table S2). Compared to older NUPTs, young NUPTs are prone to be methylated regardless of the methylation level of flanking regions (Fig. 3). The gene contents and distance from nearest gene were unchanged when comparing among NUPTs with different genetic distances (Fig. S4). Furthermore, there were weak correlation between the DNA methylation level of NUPTs and TE abundance in their flanking regions (Fig. 7). In *A*. *thaliana*, the DNA methylation could be *de novo* induced without accumulation of siRNA, possibly associated with the enrichment of histone methylation^10,38^. In *A*. *thaliana*, the young TEs also showed higher level of DNA methylation compared to that of older TEs. Such homology–dependency was observed in both TEs with and without 24–nt small RNAs^36^. A homology-dependent DNA methylation machinery could also act on NUPTs in addition to the maintenance methylation and RdDM.

In this study, we revealed a strong mutation bias of C/G to T/A transition in NUPTs of 17 species analyzed, possibly caused by DNA methylation and subsequent deamination of cytosine residues. The bias was associated with the genetic distances between plastid genome sequences and plastid DNA fragments in nuclear genomes. The results suggested that the transferred plastid DNA fragments are DNA methylated in nuclear genomes by common mechanisms for all species analyzed here. The non-CpG DNA methylation on genes probably transferred recently from organellar to nuclear genomic sequences were also observed in the study of wide range of angiosperms^39^. The DNA methylation on NUPTs is thought to be mainly dependent on the methylation maintenance machinery, while some NUPTs are more affected by RdDM machinery. Some observations may imply another mechanism of homology-dependent DNA methylation against NUPTs, though the detail is obscure in this study. The DNA methylation on NUPTs may maintain the stability of plant nuclear genomes against the integration of organellar DNA fragments and contribute to the symbiosis of nuclear and organelle genomes. In the future, the mechanism that recognize and methylate the integrated NUPTs and their influence on the plant gene and genome evolution during the symbiosis of plastids should be investigated to reveal the whole figure of the regulation of organellar DNA-like sequences and evolutionary dynamics of the plant genomes.

## Methods

### Identification of plastid DNA in plant genomes

For analyses of molecular evolution, genome sequence data of 17 plants from publicly available database (NCBI: http://www.ncbi.nlm.nih.gov/genome, Phytozome: http://www.phytozome.net, and other genome consortium’s databases) were used in this study (Table S1). To find homologous regions between nuclear and plastid genome sequences, program *blastn* implemented in BLAST ver.2.6.0 (https://blast.ncbi.nlm.nih.gov/) were used with default condition. Whole plastid genome sequences were used as queries for BLASTN search. BLAST hits with 100 bp or longer in length were collected as NUPT candidates. BLAST hits were manually inspected to remove completely identical/overlapped sequences. BLAST hits highly diverged from plastid sequences could be false positive of NUPTs. As we described later, we calculated AT transversion-based genetic distance as an indicator of homology between plastid and nuclear genomes. BLAST hits with the genetic distance larger than 0.06 were removed from NUPT candidates. Because some regions of plastid genomes have similarity to mitochondrial genomes, the origin of BLAST hits similar to such regions remains ambiguous. For 10 species with both plastid and mitochondrial genome sequences, plastid genome sequences similar to mitochondrial genomes were identified by BLASTN search between two organellar genomes. NUPT candidates homologous to such sequences were regarded as ambiguous sequences and were filtered out from the data set. The amount of ambiguous BLAST hits removed from the NUPT candidates were shown in Table S2. Finally, the remaining NUPT candidates were considered as NUPTs in this study. Because the key results of the following analysis were unchanged for all 10 species by the filtering of ambiguous sequences (Table S2, Fig. S1), the unfiltered data were used for the analysis of nucleotide changes in the remaining 7 species.

### Molecular Evolutionary analyses

Nucleotide changes between plastid genomes and NUPTs were identified. Because plant organellar genomes have low mutation rate compared with nuclear genomes^40–42^, we assumed majority of nucleotide substitutions had occurred in NUPTs. Based on this assumption, each substitution was classified into four categories: A/T to C/G transversions, C/G to A/T transversions, T/A to C/G transitions, and C/G to T/A transitions. Nucleotide substitutions were identified from the aligned sequences of BLAST search. Compositions of GC contents were also calculated for plastid DNAs and NUPTs. Alignment gaps and ambiguous nucleotides were removed from the aligned sequences and were not counted.

Genetic distances between plastid sequences and NUPTs were calculated to estimate relative integration time of NUPTs. Our results suggested biased direction of nucleotide substitutions from C/G to T/A transitions putatively caused by cytosine DNA methylation and subsequent deamination. Thus, we used transversions between adenine (A) and thymine (T) that could have low effect by biased mutation to estimate genetic distances instead of p-distances calculated by all substitutions. This AT transversion-based genetic distance was calculated by following formula:

$${d}_{AT}=\frac{({N}_{A\to T}+{N}_{T\to A})}{{L}_{align}}$$

in which *N*_AT_ and *N*_TA_ represent A to T and T to A nucleotide change between plastid and nuclear sequences, *L*_align_ represents alignment sequence length without alignment gaps.

### Levels of DNA methylation

We analyzed intensively sequenced DNA methylation data of 2 plants, *Arabidopsis thaliana*^43^ and *Oryza sativa*^44^ for analyses of CpG and non–CpG methylation within NUPTs. The mapped data of both species in previous study^45^, were used in this study. In brief, the reference genomes (*A*. *thaliana*; Release 10 of the Arabidopsis Information Resources, *O*. *sativa*; Release 7 of the MSU Rice Genome Annotation Project) were processed to convert C/G to T/A for mapping of methylome data. The sequence data of both species (*A*. *thaliana*; GSE10966, *O*. *sativa*; GSE22591) were downloaded from Gene Expression Omnibus (GEO, available in: https://www.ncbi.nlm.nih.gov/geo/) and were mapped to the converted reference genomes by program Bowtie^46^ under conditions described^47^. Mapped reads were extracted and counted for each NUPT. For each NUPT, weighted methylation level^48^ was calculated by following formula:

$${M}_{weighted}=\frac{{\sum }_{{\rm{i}}=1}^{{\rm{n}}}{{\rm{C}}}_{{\rm{i}}}}{{\sum }_{i=1}^{n}({C}_{{\rm{i}}}+{{\rm{T}}}_{{\rm{i}}})}$$

in which C_i_ and T_i_ represent methylated (C_*i*_) and unmethylated (T_i_) cytosine counts of the i_th_ cytosine site. NUPTs with no methylome data were excluded from the following analyses. Age distributions of CpG and non–CpG methylations were analyzed based on the genetic distance between plastid sequences and NUPTs. To analyze the effect of the status of adjacent regions on NUPTs, the level of cytosine methylations within flanking sequences was analyzed and correlations with methylation status of NUPTs were examined. Whole genome profiling of cytosine methylations reported in 86 *A*. *thaliana* mutant lines^31^ were used to survey the methylation status of NUPTs and to detect the factors involved in DNA methylation of these sequences. Single base-pair methylation data (GSE39901) were downloaded from GEO and were used for the analysis. To estimate the methylation pattern of *O*. *sativa ddm1a/1b*, *drm2* mutants^32,33^, whole genome bisulfite-seq data (GSE108527, GSE81436) were downloaded form GEO. Sequence data were trimmed by Trimmomatic ver.0.36^49^ and trimmed reads were mapped to reference genome by program *bismark* in Bismark suite ver. 0.14.4^50^ using default parameters. Mapped reads were counted by program *bismark_methylation_extractor* implemented in Bismark suite.

### TE and gene abundance in flanking regions of NUPTs

The relationship between feature of inserted site and NUPTs methylation level was analyzed using TAIR10 annotation information for *A*. *thaliana* and annotation information in Release 7 of the MSU Rice Genome Annotation Project for *O*. *sativa*. The number of genes in 50 Kb of each flanking region (in total 100 Kb region for each NUPT) and distance from nearest gene to NUPTs 5′ or 3′ end position were surveyed. The number of TEs in 5 Kb of each flanking region (in total 10 Kb region) was counted and correlation with DNA methylation level was tested.

### Survey of genes responsible for DNA methylation of NUPTs

To find the candidate genes responsible for DNA methylation of NUPTs, the screen of *A*. *thaliana* mutant lines treated by ethyl methanesulfonate (EMS)^45,51^ was conducted. For screening, PCR primers for four organellar DNA-like fragments were designed. gDNA extracted from EMS mutant lines were digested by endonuclease McrBC which cleaves DNAs containing methylcytosine. If organellar DNA-like fragments are hypomethylated, PCRs with McrBC-digested gDNAs will amplify these hypomethylated DNAs. Multiplex PCR of 4 target regions were conducted.

## Supplementary information

Supplemental Figures
