# Author Correction: Genome defense against integrated organellar DNA fragments from plastids into plant nuclear genomes through DNA methylation

**DOI:** 10.1038/s41598-020-67805-w

**Published:** 2020-06-25

**Authors:** Takanori Yoshida, Hazuka Y. Furihata, Taiko Kim To, Tetsuji Kakutani, Akira Kawabe

**Affiliations:** 1 Faculty of Life Science, Kyoto Sangyo University, Kyoto, Kyoto Japan; 2 Faculty of Science, The University of Tokyo, Bunkyo-ku, Tokyo, Japan; 3 Department of Integrated Genetics, National Institute of Genetics, Mishima, Shizuoka Japan; 4 Department of Genetics, School of Life Science, The Graduate University for Advanced Studies (SOKENDAI), Mishima, Shizuoka Japan

Correction to: *Scientific Reports* 10.1038/s41598-019-38607-6, published online 14 February 2019

In the Supplementary Information file originally published with this Article, Supplementary Tables (Tables S1-4) were omitted. These errors have been corrected in the Supplementary Information that now accompanies the Article.

