# Supplementary material for: Genome defense against integrated organellar DNA fragments from plastids into plant nuclear genomes through DNA methylation
Source: Sci Rep. 2019 Feb 14;9:2060. doi: 10.1038/s41598-019-38607-6 (PMC6376042; doi:10.1038/s41598-019-38607-6)
Supplement: Supplementary file 1 — Supplemental Figures [file 41598_2019_38607_MOESM1_ESM.pdf]

Supplementary Information

Genome defense against integrated organellar DNA fragments from plastids into plant nuclear genomes through DNA methylation

Takanori Yoshida[1], Hazuka Y. Furihata[1], Taiko Kim To[2], Tetsuji Kakutani[2,3,4], and Akira Kawabe[1*]

[1] Faculty of Life Science, Kyoto Sangyo University, Kyoto, Kyoto, Japan
[2] Faculty of Science, The University of Tokyo, Bunkyo-ku, Tokyo, Japan
[3]Department of Integrated Genetics, National Institute of Genetics, Mishima, Shizuoka, Japan
[4]Department of Genetics, School of Life Science, The Graduate University for Advanced Studies (SOKENDAI), Mishima, Shizuoka, Japan

* akiraka@cc.kyoto-su.ac.jp

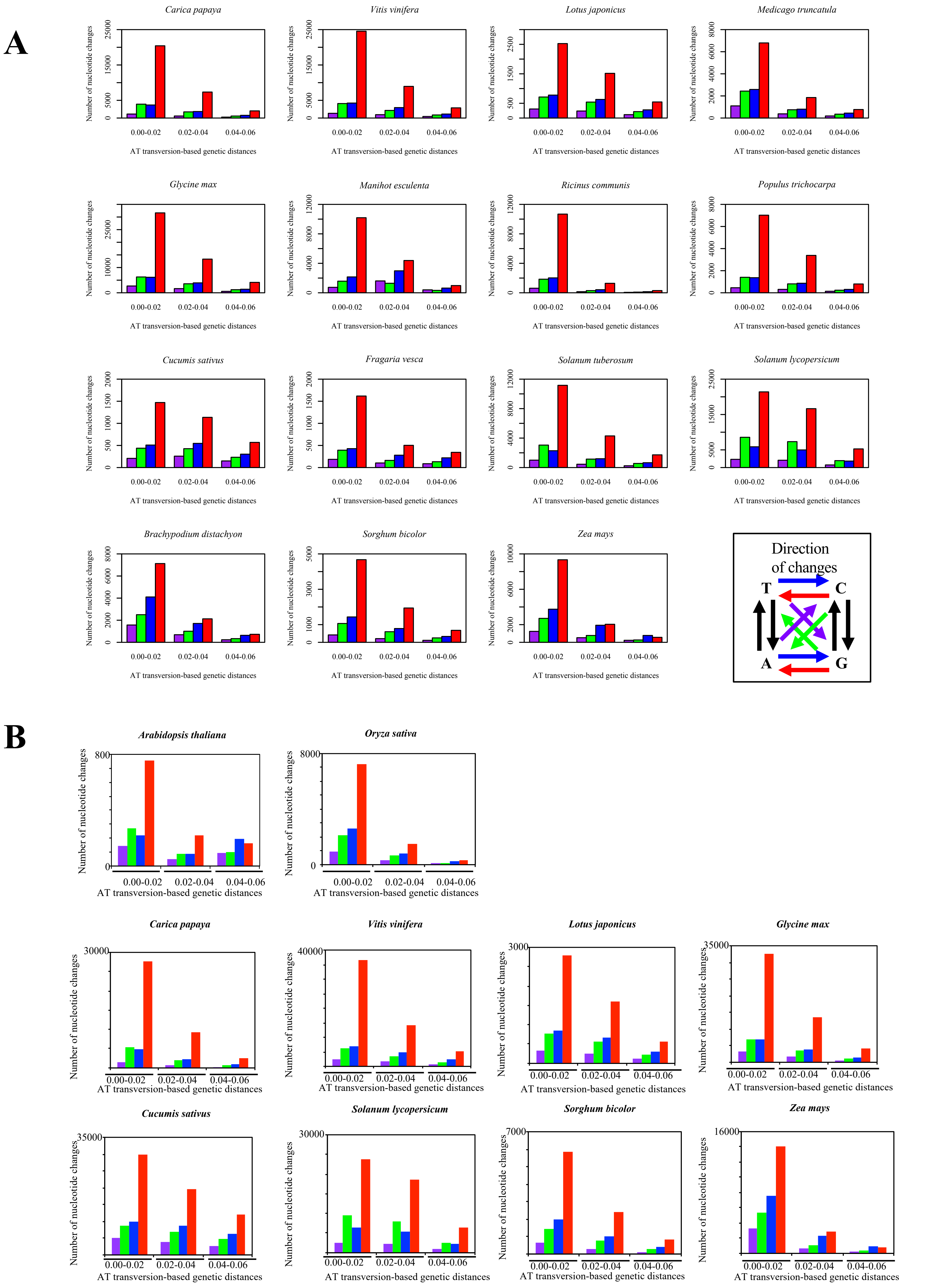

Figure S1. Age distribution of segregating sites for 15 species. (A) Nucleotide change between NUPTs and corresponding plastid DNA sequences were shown for 15 species. NUPTs were grouped by genetic distance. For 8 species (*C.papaya*, *V. vinifera*, *L. japonicus*, *G. max*, *C. sativus*, *S. lycopersicum*, *S. bicolor*, *Z. mays*), ambiguous NUPTs that also have similarity with mitochondrial DNA were removed. **(B)** For 10 species, data without filtering of ambiguous NUPTs were also shown.

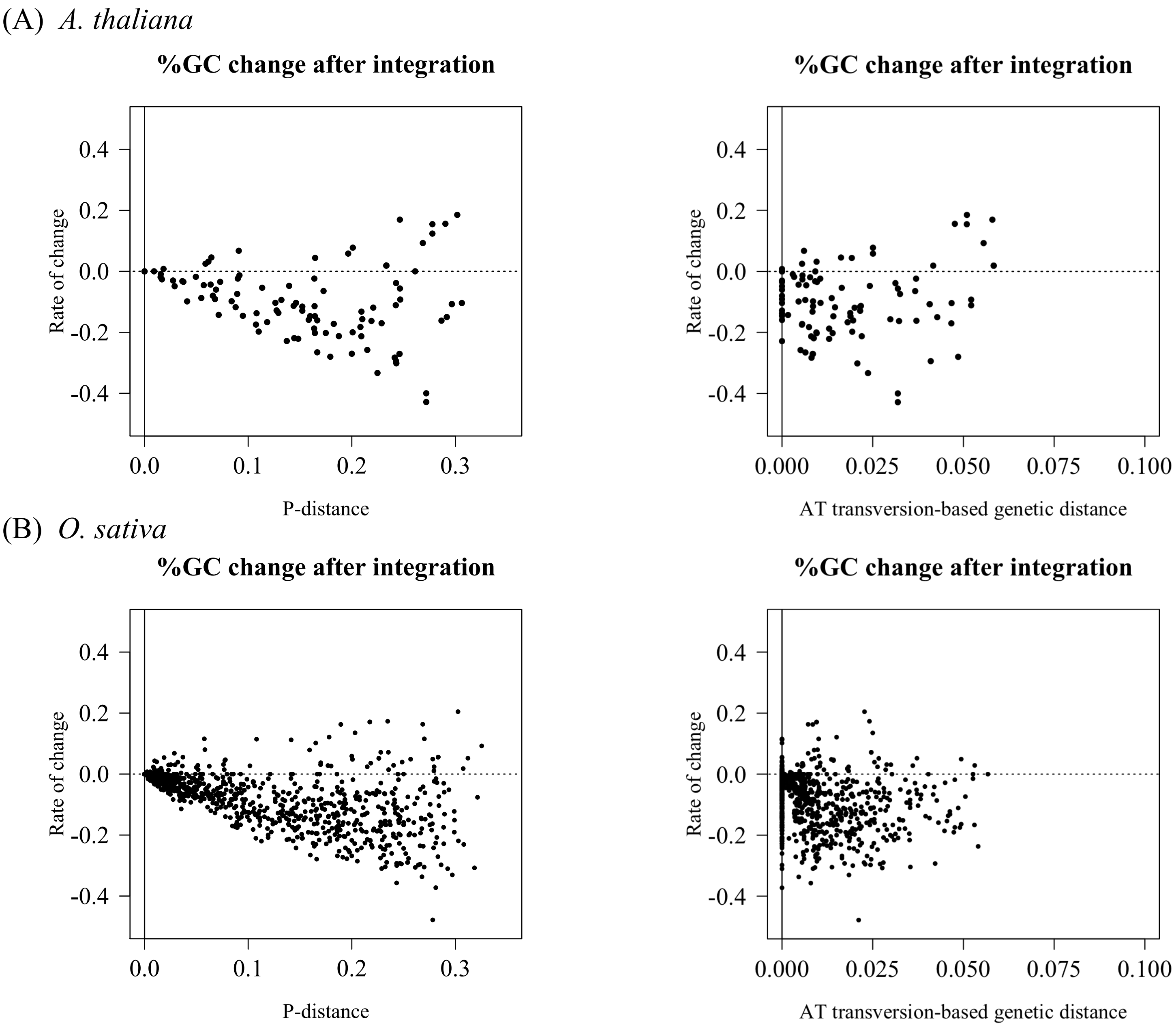

Figure S2. Scatter plot of differences of G/C residues compositions. Changes of GC contents were calculated from GC composition of NUPTs and corresponding plastid DNA sequences. Horizontal line represents genetic distance between NUPTs and plastid DNA sequences. Vertical line represents change of GC contents in NUPTs.

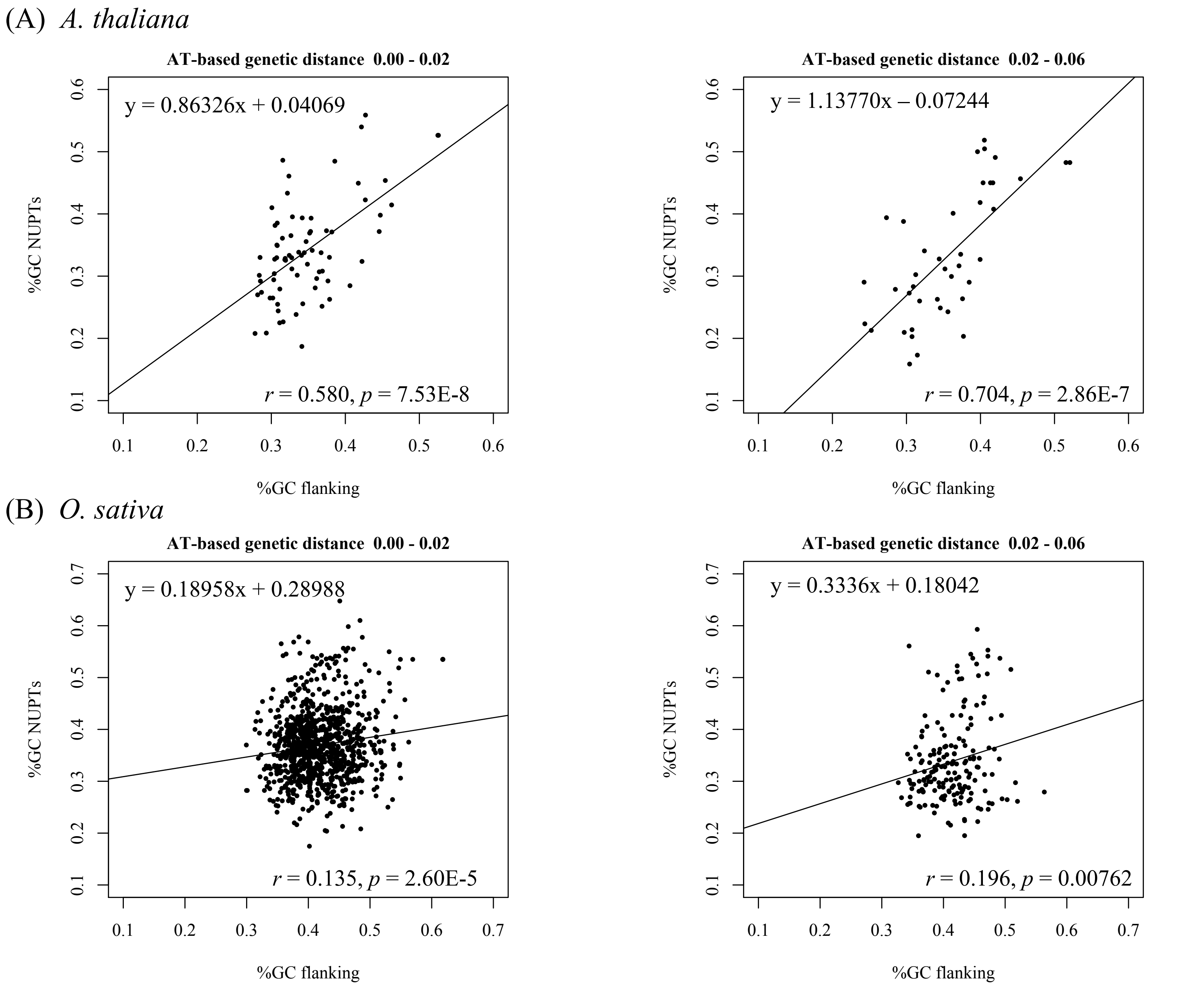

Figure S3. Relationship between GC contents of NUPT and its flanking region. Correlations between NUPTs and its adjacent sequences (1 Kb each) from both 5' and 3' flanking region were shown. *r*; Pearson correlation coefficient, *p*; *p*-value of the test for correlation.

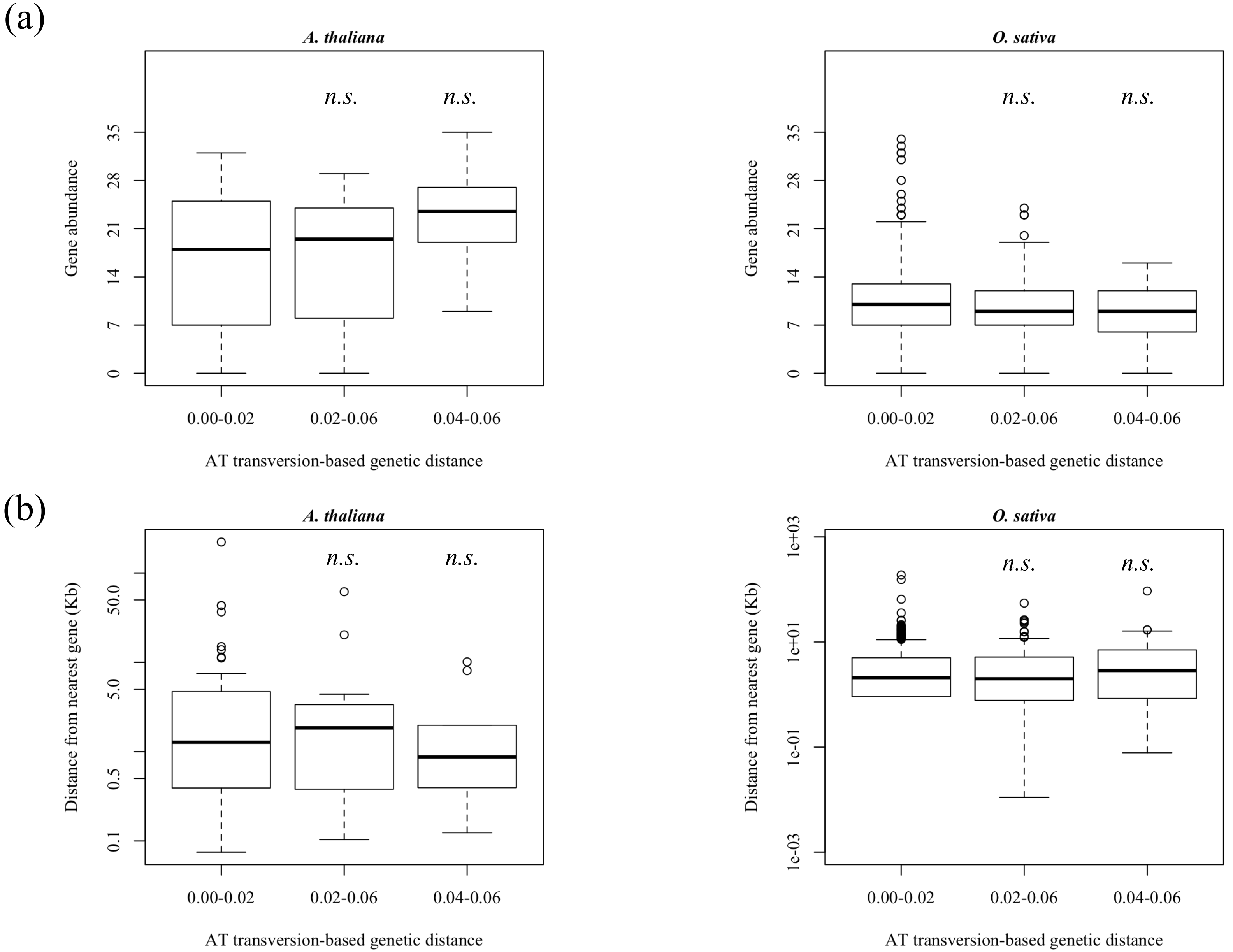

Figure S4. Characteristics of integrated regions. (A) Gene abundance in flanking region of NUPTs. Protein coding genes in 50 Kb sequences from both 5' and 3' flanking regions were counted. Vertical line represents gene abundance (number of gene/flanking region). Bold lines represent medians. Upper and lower lines of boxes represent the first and third quartiles. Circles represent outliers. (B) Logarithmic distance from nearest-neighbour gene. Distances from 5' or 3' terminal of NUPTs to nearest-neighbour genes were measured. Vertical line represents logarithm of distance (log[Kb]). Wilcoxon rank sum test: *n.s.*; not significant.

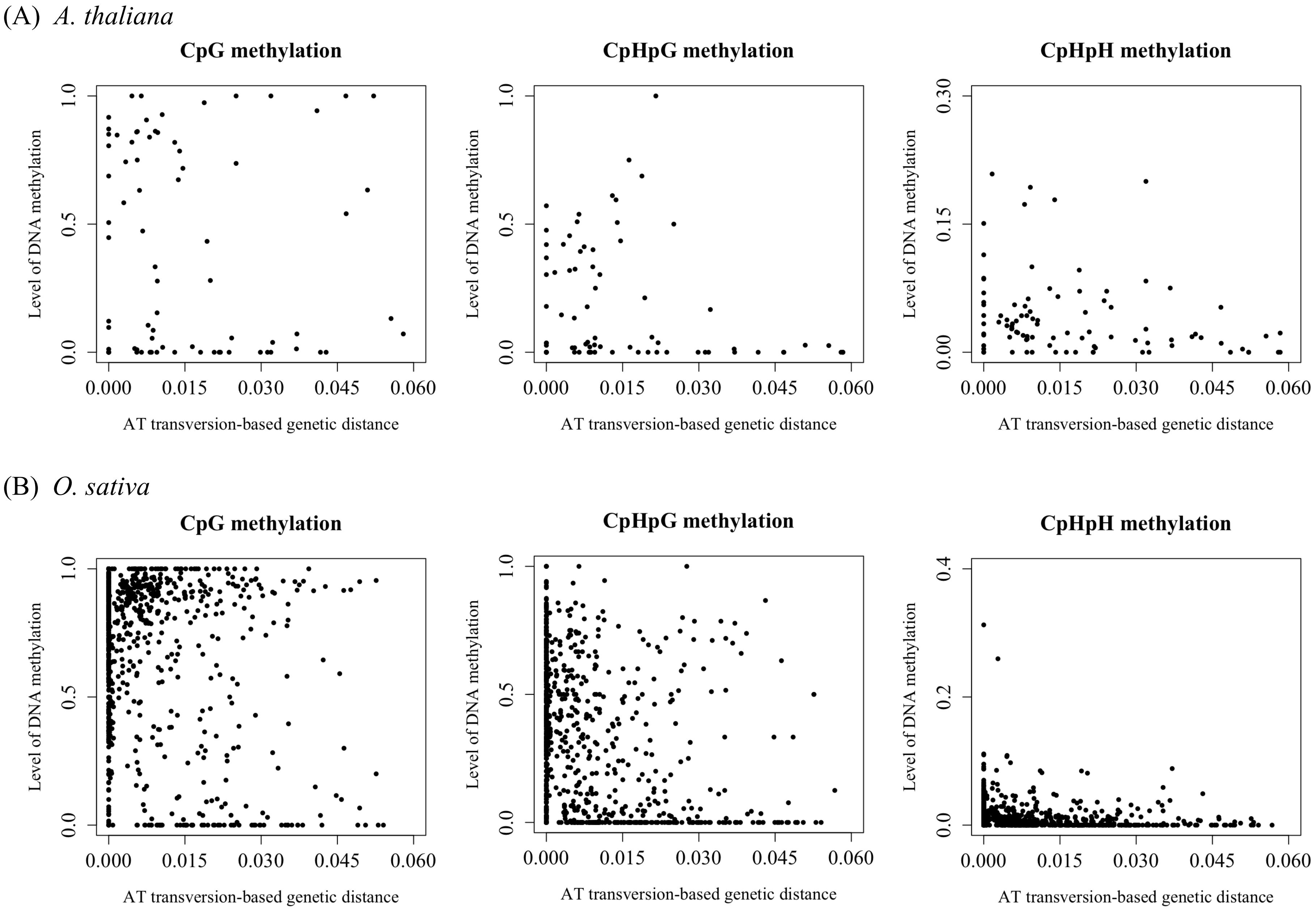

Figure S5. Scatter plot of levels of CpG and non-CpG methylations. Horizontal line represents AT transversion-based genetic distance between NUPTs and corresponding plastid DNA sequences. Vertical line represents level of DNA methylation.

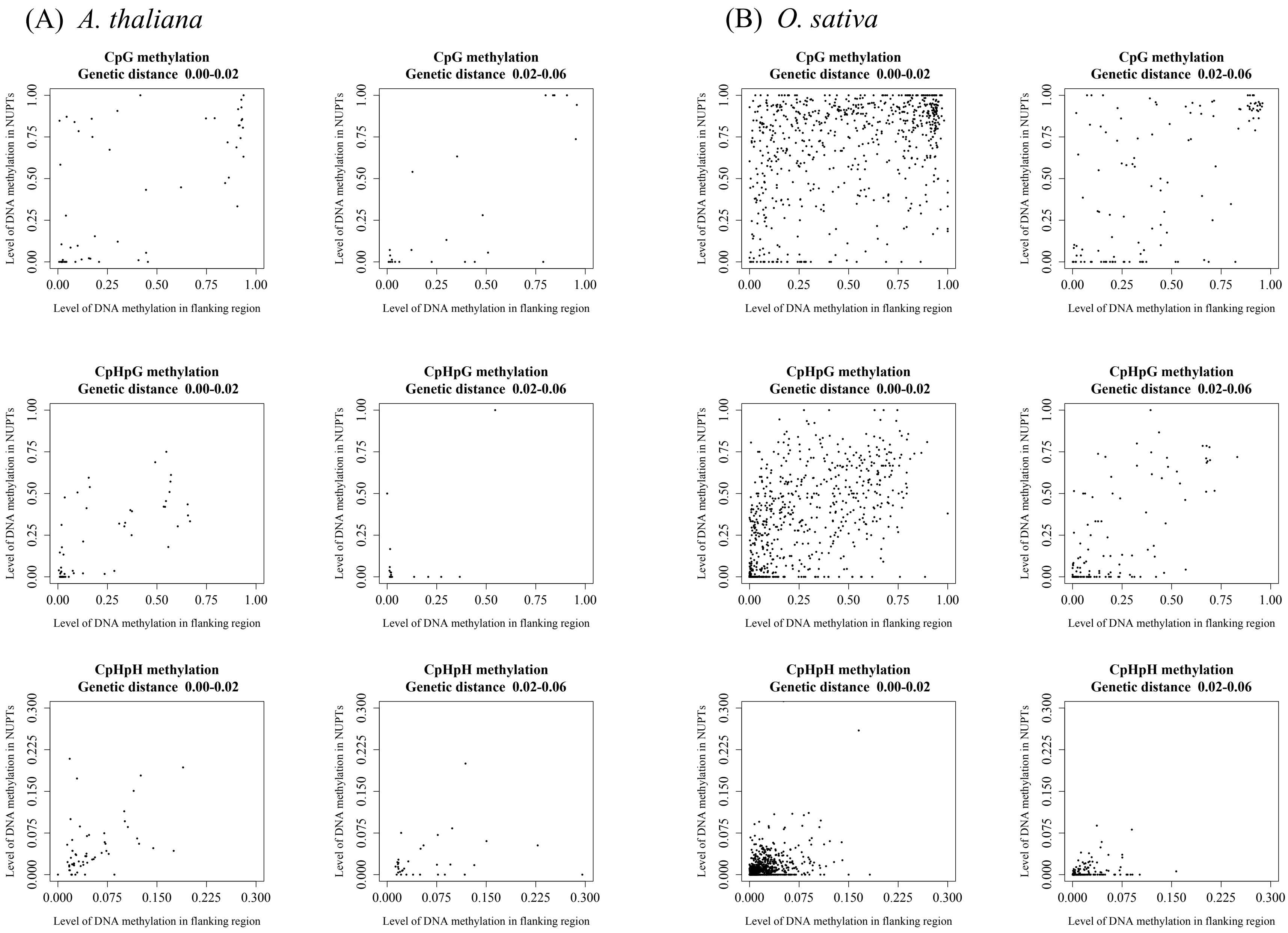

Figure S6. DNA methylation levels of NUPTs and their flanking regions. Horizontal line represents the level of DNA methylation in flanking regions. Vertical line represents the level of DNA methylation in NUPTs. For each species, plots in the left side show the data of younger aged NUPTs, while plots in the right side show the data of middle and older aged NUPTs.

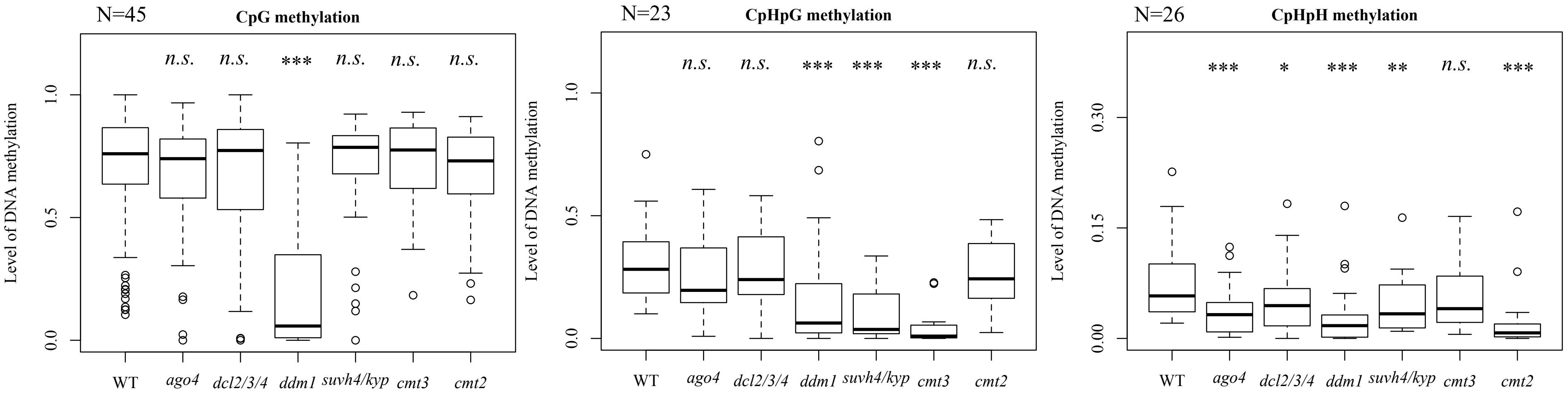

Figure S7. Boxplot of DNA methylation level in epigenetic mutant lines. Levels of DNA methylation in WT and mutant lines (*ago4*, *dcl2/3/4*, *ddm1*, *suvh4/kyp*) were shown. Plotted NUPTs were filtered out by their WT methylation level to eliminate unmethylated/unmapped sequences (CpG methylation level > 0.1, CpHpG methylation level > 0.1, CpHpH methylation level > 0.02). Tick lines represents medians. Outliers were shown by white circles. Wilcoxon rank sum test: *; $p < 0.05$, **; $p < 0.01$, ***; $p < 0.001$, *n.s.*; not significant.

Supplementary Table S1 Genomic data used in this study

Taxon	Source	Assembly / Accession number / bulk data file name			Data collection date
		nucleus	plastid	mitochondrion	
**Eudicots:**					
Arabidopsis thaliana	GenBank	NC_003070.9, NC_003071.7, NC_003074.8, NC_003075.7, NC_003076.8	NC_000932.1	NC_037304.1	12-Apr-10, 18-Aug-18
Carica papaya	Phytozome	Cpapaya_113	EU431223.1	NC_012116.1	20-May-12
Vitis vinifera	Phytozome	Vvinifera_145	DQ424856.1	NC_012119.1	22-May-12
Lotus japonicus	Kazusa DNA Research Institute, http://www.kazusa.or.jp/lotus/	lotus_r2.5	NC_002694.1	NC_016743.2	18-Aug-18
Medicago truncatula	GenBank	MedtrA17_3.5	NC_003119.6		13-Jun-12
Glycine max	GenBank	Glycine max assembly V1.0	DQ317523.1	NC_020455.1	13-Jun-12
Manihot esculenta	Phytozome	Mesculenta_147	NC_010433.1		13-Jul-12
Ricinus communis	GenBank	JCVI_RCG_1.1	JF937588.1		8-Jun-12
Populus trichocarpa	GenBank	Poptr1_1	NC_009143.1		8-Jun-12
Cucumis sativus	GenBank	CSB10A_v1	NC_007144.1	NC_016005.1	21-Mar-13
Fragaria vesca	Genome Database for Rosaceae, http://www.rosaceae.org/	Whole Genome Assemblies (v1.1)	NC_015206.1		13-Jul-12
Solanum tuberosum	Solanaceae Genomics Resource, http://solanaceae.plantbiology.msu.edu/	PGSC_DM_v3_scaffolds	DQ386163.2		13-Jul-12
Solanum lycopersicum	The International Tomato Genome Sequencing Consortium, http://solgenomics.net/organism/Solanum_lycopersicum/genome	S_lycopersicum_chromosomes.2.40	NC_007898	NC_035963.1	13-Jul-12
**Monocots:**					
Brachypodium distachyon	GenBank	NC_16131.1 - NC_16135.1	EU325680.1		9-May-13
Oryza sativa	GenBank	NC_008394- NC_008405	GU592207.1	NC_011033.1	16-May-12
Sorghum bicolor	GenBank	NC_012870.1- NC_012879.1 NW_002994219.1- NW_002994226.1	EF115542.1	NC_008360.1	18-Apr-12
Zea mays	Phytozome	Zmays_181	X86563.2	NC_007982.1	22-May-12

Supplementary table S2 Summary of nucleotide change between NUPTs and their original plastid DNAs

Species	Distance[a]	N[b]	Length[c]	GC composition (without filtering)		ptDNA NUPTs	Transition (without filtering) AT GC		GC AT		Ratio (without filtering)[d]	
Arabidopsis thaliana	0.00-0.02	63 (10)	22370 (3611)	0.324	(0.333)		184	(222)	699	(759)	0.26	(0.29)
	0.02-0.04	20 (1)	4092 (181)	0.312	(0.311)		87	(90)	216	(221)	0.40	(0.41)
	0.04-0.06	17 (1)	3175 (147)	0.388	(0.389)		189	(193)	156	(162)	1.21	(1.19)
Carica papaya	0.00-0.02	1022 (333)	386499 (104195)	0.327	(0.325)		3681	(4747)	20422	(27675)	0.18	(0.17)
	0.02-0.04	308 (77)	78969 (17521)	0.280	(0.278)		1838	(2265)	7346	(9240)	0.25	(0.25)
	0.04-0.06	104 (24)	22308 (4739)	0.272	(0.272)		761	(885)	2017	(2494)	0.38	(0.35)
Vitis vinifera	0.00-0.02	1352 (899)	410124 (243250)	0.341	(0.331)		4232	(6956)	24559	(36481)	0.17	(0.19)
	0.02-0.04	508 (344)	113339 (75521)	0.308	(0.303)		2950	(4943)	8946	(14111)	0.33	(0.35)
	0.04-0.06	181 (162)	37392 (32263)	0.305	(0.3)		1123	(2268)	2874	(5285)	0.39	(0.43)
Lotus japonicus	0.00-0.02	488 (46)	161457 (7641)	0.359	(0.366)		778	(839)	2526	(2790)	0.31	(0.3)
	0.02-0.04	157 (8)	29170 (1209)	0.312	(0.318)		631	(655)	1516	(1593)	0.42	(0.41)
	0.04-0.06	68 (1)	11294 (242)	0.304	(0.31)		277	(292)	545	(562)	0.51	(0.52)
Medicago truncatula	0.00-0.02	1065	998669	0.337	-		2585	-	6795	-	0.38	-
	0.02-0.04	192	48498	0.306	-		801	-	1854	-	0.43	-
	0.04-0.06	93	16166	0.305	-		444	-	770	-	0.58	-
Glycine max	0.00-0.02	2770 (133)	693076 (18906)	0.301	(0.306)		6140	(6889)	31633	(32713)	0.19	(0.21)
	0.02-0.04	1024 (11)	210231 (1892)	0.262	(0.264)		3927	(3958)	13333	(13564)	0.29	(0.29)
	0.04-0.06	328 (2)	57773 (512)	0.263	(0.265)		1411	(1420)	4120	(4181)	0.34	(0.34)
Manihot esculenta	0.00-0.02	508	158956	0.316	-		2153	-	10189	-	0.21	-
	0.02-0.04	183	82796	0.315	-		2982	-	4383	-	0.68	-
	0.04-0.06	59	17956	0.266	-		639	-	981	-	0.65	-
Ricinus communis	0.00-0.02	824	327801	0.331	-		2023	-	10680	-	0.19	-
	0.02-0.04	105	24351	0.275	-		422	-	1286	-	0.33	-
	0.04-0.06	33	6468	0.229	-		152	-	300	-	0.51	-
Populus trichocarpa	0.00-0.02	395	136386	0.317	-		1366	-	7022	-	0.19	-
	0.02-0.04	177	44982	0.282	-		864	-	3383	-	0.26	-
	0.04-0.06	59	11080	0.279	-		307	-	798	-	0.38	-
Cucumis sativus	0.00-0.02	171 (161)	45352 (39515)	0.330	(0.349)		509	(982)	1471	(2972)	0.35	(0.33)
	0.02-0.04	97 (63)	21750 (11411)	0.293	(0.307)		547	(855)	1136	(1944)	0.48	(0.44)
	0.04-0.06	46 (42)	8832 (8486)	0.277	(0.309)		303	(623)	569	(1190)	0.53	(0.52)
Fragaria vesca	0.00-0.02	289	71199	0.365	-		428	-	1618	-	0.26	-
	0.02-0.04	68	10950	0.349	-		280	-	503	-	0.56	-
	0.04-0.06	41	6511	0.355	-		220	-	344	-	0.64	-
Solanum tuberosum	0.00-0.02	879	494977	0.373	-		2284	-	11162	-	0.20	-
	0.02-0.04	297	59742	0.314	-		1198	-	4294	-	0.28	-
	0.04-0.06	121	21945	0.328	-		657	-	1725	-	0.38	-
Solanum lycopersicum	0.00-0.02	1978 (280)	762194 (53788)	0.356	(0.36)		5871	(6393)	21399	(23902)	0.27	(0.27)
	0.02-0.04	969 (118)	308531 (26794)	0.328	(0.333)		4986	(5397)	16652	(18531)	0.30	(0.29)
	0.04-0.06	402 (65)	73683 (14066)	0.304	(0.32)		1834	(2216)	5272	(6306)	0.35	(0.35)
Brachypodium distachyon	0.00-0.02	1961	1101568	0.378	-		4104	-	7126	-	0.58	-
	0.02-0.04	293	65733	0.346	-		1707	-	2125	-	0.80	-
	0.04-0.06	104	19002	0.339	-		634	-	726	-	0.87	-
Oryza sativa	0.00-0.02	740 (220)	853516 (61497)	0.387	(0.387)		2054	(2628)	5721	(7253)	0.36	(0.36)
	0.02-0.04	117 (25)	24132 (3938)	0.342	(0.344)		700	(829)	1288	(1492)	0.54	(0.56)
	0.04-0.06	27 (6)	4446 (870)	0.311	(0.324)		170	(224)	253	(295)	0.67	(0.76)
Sorghum bicolor	0.00-0.02	617 (181)	191086 (40640)	0.367	(0.371)		1435	(1962)	4674	(5845)	0.31	(0.34)
	0.02-0.04	159 (35)	33293 (7516)	0.324	(0.328)		780	(984)	1941	(2381)	0.40	(0.41)
	0.04-0.06	62 (13)	11451 (2243)	0.302	(0.306)		333	(397)	678	(809)	0.49	(0.49)
Zea mays	0.00-0.02	1316 (930)	919584 (233513)	0.375	(0.387)		3748	(7561)	9337	(14124)	0.40	(0.54)
	0.02-0.04	347 (62)	60177 (14351)	0.306	(0.324)		1933	(2267)	2041	(2825)	0.95	(0.8)
	0.04-0.06	123 (20)	18801 (4582)	0.291	(0.312)		777	(947)	563	(849)	1.38	(1.12)

For 10 species (*A. thaliana*, *C.papaya*, *V. vinifera*, *L. japonicus*, *G. max*, *C. sativus*, *S. lycopersicum*, *O. sativa*, *S. bicolor*, *Z. mays*), ambiguous NUPTs that also have similarity with mitochondrial DNA were removed.

a Distance between plastid DNA and NUPTs based on transversional change between A and T.

b Number of NUPTs. Figures in parentheses are ambiguous BLAST hits removed from the NUPT data sets.

c Cumulative length of NUPTs. Figures in parentheses were those of ambiguous BLAST hits removed from the NUPT data sets.

d Ratio of (A/T to G/C) transitions to (C/G to T/A) transitions.

Supplementary Table S3 Location of highly methylated plastid DNA fragments with high genetic distance on the nuclear genomes

No.	CpG methylation level	Genetic distance	Location	notation
745	1	0.0466	Chr3:7859381..7859530	Beside AT3G22237 (pseudogene),<br>2.5Kb from AT3G22238 (pseudogene),<br>4Kb from AT3G22240 (protein coding),<br>3.5Kb from AT3G22235 (protein coding)
747	0.5405	0.0467	Chr3:20595867..20596084	Within intron of AT3G55530
12	0.6329	0.0509	Chr5:2827527..2827742	Within exon of AT5G08690
732	1	0.0521	Chr3:7848620..7848848	1Kb from AT3G22232 (pseudogene)
989	0.9423	0.041	Chr2:6375473..6375596	2.5Kb from AT2G14840 (pseudogene)<br>3Kb from AT2G14843 (transposable element gene)

Supplementary Table S4 Location of plastid DNA fragments that decrease DNA methylation on RNAi mutants, *ago4* and *dcl2/3/4*

No.	Genetic distance	Location	notation
1	0.001652893	Chr5:8133439..8134044	2.3 Kb from AT5TE29440
472	0	Chr1:11552177..11552312	Beside AT1TE37395
			1Kb from AT1TE37390
			1.8Kb from AT1TE37385
			2.5Kb from AT1TE37380
